# Supplementary material for: Accelerated HF-rTMS Modifies SERT Availability in the Subgenual Anterior Cingulate Cortex: A Canine [11C]DASB Study on the Serotonergic System
Source: J Clin Med. 2022 Mar 10;11(6):1531. doi: 10.3390/jcm11061531 (PMC8950510; doi:10.3390/jcm11061531)
Supplement: Supplementary file 1 [file jcm-11-01531-s001.zip › jcm-1585515-supplementary.pdf]

Supplemental materials

Supplemental Table S1. Statistics for contrasts within three treatment groups.

	**5 Sessions Active - 20 Sessions Sham**				**20 Sessions Active – 20 Sessions Sham**				**5 Sessions Active - 20 Sessions Active**			
	Estimate	SE	z-ratio	*p*-value	Estimate	SE	z-ratio	*p*-value	Estimate	SE	z-ratio	*p*-value
T0	-0.2561	0.204	-1.254	0.6295	0.2675	0.215	1.243	0.6421	0.0115	0.160	0.072	1.0000
T1	-0.2247	0.216	-1.038	0.8978	-0.0160	0.219	-0.073	1.0000	-0.2407	0.163	-1.475	0.4206
T2	-0.1745	0.203	-0.861	1.0000	-0.0535	0.211	-0.254	1.0000	-0.2280	0.152	-1.503	0.3982
T3	-0.3398	0.194	-1.748	0.2415	0.3096	0.204	1.515	0.3892	-0.0302	0.152	-0.199	1.0000

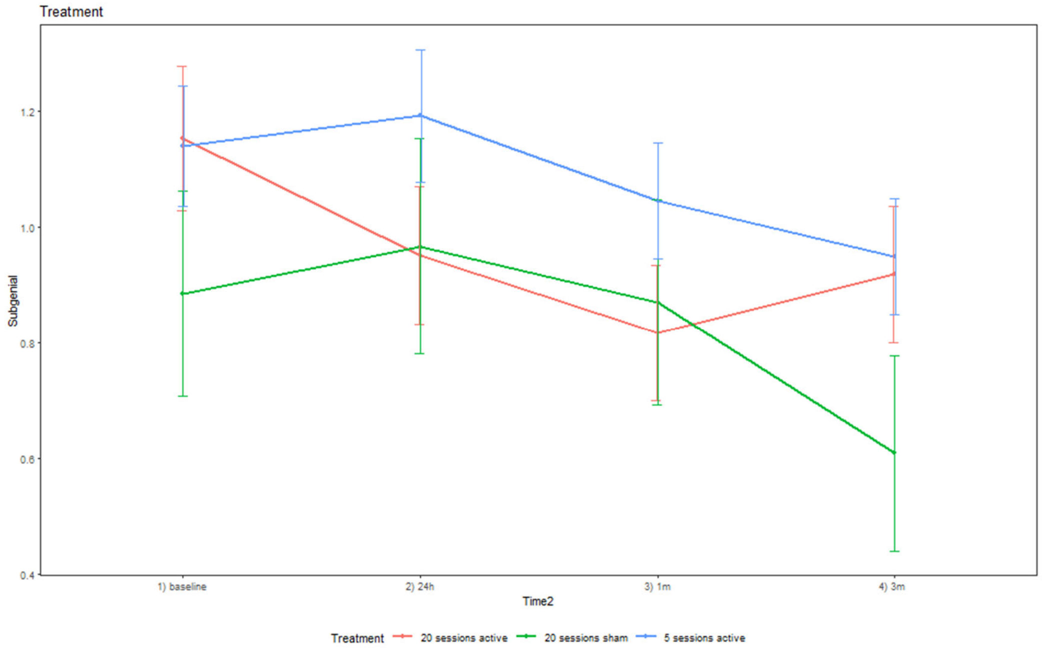

Supplemental Figure S1. Line plot for sgACC for three treatment groups at each individual time moment. SE's are displayed as error bars.
